# Antibacterial and Anticancer Potential of *Alhagi maurorum* Ethanol Crude Extract: LC-MS-Guided Evidence and In Silico Mechanistic Insights

**DOI:** 10.3390/ijms27114766

**Published:** 2026-05-25

**Authors:** Ibrahim Mahmood Mahdi, Ahmed Abdul Kareem Najm

**Affiliations:** 1 Department of Biology, College of Science, Al Rafidain University, Baghdad 10064, Iraq; 2 Department of Food Science, Faculty of Science and Technology, Universiti Kebangsaan Malaysia, Bangi 43600, Malaysia

**Keywords:** *ADME*, *Alhagi maurorum*, in silico *toxicity*, molecular docking

## Abstract

The worldwide rise in antimicrobial resistance, along with the ongoing prevalence of cancer, underscores the pressing need for novel, safe, and multifunctional therapeutic candidates. Medicinal plants continue to serve as a valuable source of chemically diverse bioactive molecules that modulate multiple biological targets. In this investigation, the preliminary screening of the antibacterial and anticancer activities of an ethanolic extract of *Alhagi maurorum* (*A. maurorum*) was comprehensively evaluated using integrated chemical characterization, in vitro bioassays, and in silico approaches. A liquid chromatography–mass spectrometry (LC-MS) analysis demonstrated a rich phytochemical profile including glucosinolates, phenolic acids, gallotannins, fatty acids, alkaloids, carotenoid derivatives, and *2-hexyldecanoic acid*-associated constituents. Antibacterial efficacy was assessed by disk diffusion and minimum inhibitory concentration (MIC) testing against Escherichia coli (*E. coli*) and Bacillus cereus (*B. cereus*), with the extract producing inhibition zones similar to those observed with streptomycin. Anticancer effects were determined using 3-(4,5-dimethylthiazol-2-yl)-2,5-diphenyltetrazolium bromide (MTT) assays with MCF-7 breast carcinoma cells and Hs27 normal fibroblasts over 24, 48, and 72 h, revealing a time-dependent, selective decrease in malignant cell viability with relatively limited toxicity towards normal cells. Induction of apoptosis was further verified by propidium iodide (PI) staining. A molecular docking analysis highlighted *2-hexyldecanoic acid* as the principal active compound, with a strong binding affinity for critical bacterial targets (GyrA, GyrB, and RpoB). In silico toxicity and *ADME* (absorption, distribution, metabolism, and excretion) assessments indicated favorable drug-like properties, good gastrointestinal uptake, and acceptable safety profiles. Altogether, these results provide combined experimental and computational support for *A. maurorum* as a promising source of dual-purpose antibacterial and anticancer agents and lay a mechanistic foundation for subsequent preclinical studies.

## 1. Introduction

The swift rise in multidrug-resistant (MDR) bacterial strains represents a critical danger to global public health, diminishing the efficacy of standard antibiotics and contributing to higher rates of illness and death worldwide [1,2]. At the same time, cancer continues to rank among the primary causes of mortality, with chemoresistance and therapy-related toxicity restricting sustained therapeutic outcomes [3]. Together, these issues have heightened scientific interest in natural products as alternative or adjunctive therapeutic resources, given their broad chemical diversity, target selectivity, and long-standing application in traditional medicinal systems [4,5].

Medicinal plants are especially appealing because they typically harbor numerous bioactive compounds that can act synergistically on various molecular targets, thereby reducing the likelihood of resistance emergence and enhancing therapeutic efficacy [6]. In particular, edible species and those long utilized in traditional remedies constitute a valuable yet under-investigated reservoir for the development of multifunctional drug candidates.

*Alhagi maurorum* L. (*A. maurorum) is a thorny perennial species widely distributed across the Middle East and* the Mediterranean basin. It has been traditionally employed for medicinal purposes, with documented antioxidant, anti-inflammatory, antimicrobial, and anticancer effects [7,8,9]. Phytochemical studies have revealed that *A. maurorum* contains diverse secondary metabolites, including flavonoids, phenolic acids, fatty acids, and glucosinolate-associated compounds, many of which exhibit bioactivities relevant to infectious diseases and cancer [10,11,12]. Nevertheless, earlier investigations have largely examined individual biological effects in isolation, lacking an integrated approach that combines comprehensive chemical characterization, mechanistic insights, and safety evaluation within a unified study design.

Accordingly, this investigation sought to provide a thorough assessment of the antibacterial and anticancer potential of A. maurorum ethanolic extract by combining liquid chromatography–mass spectrometry (LC-MS)-driven phytochemical characterization with in vitro antibacterial and cytotoxicity testing, apoptosis evaluation, molecular docking simulations, and in silico toxicity and pharmacokinetic studies. This multidisciplinary strategy was intended to enhance biological insight, identify promising lead molecules, and evaluate their translational potential.

## 2. Results

### 2.1. Liquid Chromatography–Mass Spectrometry (LC-MS) Phytochemical Profiling

**Figure 1:**
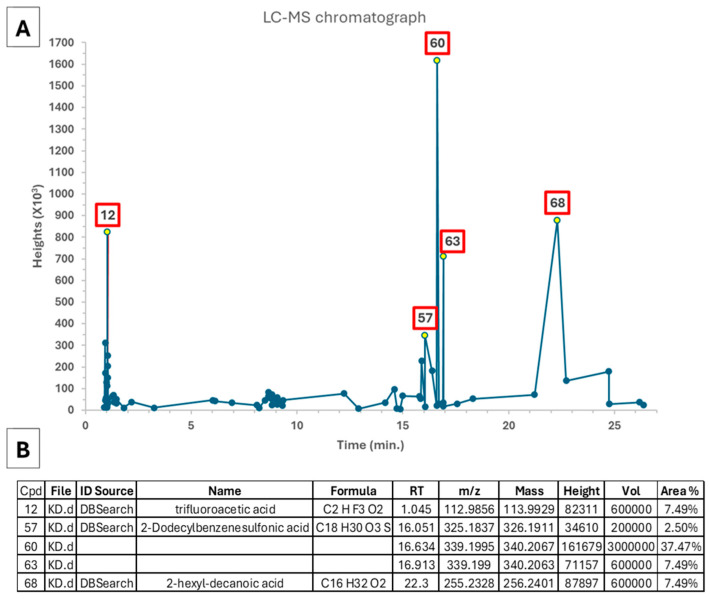
Shows the ethanol crude extracts and their LC-MS chemical profiles. (**A**) The chemograph profile showed that compounds 12, 57, 60, 63, and 68 had the highest peak intensities among the 73 detected compounds. (**B**) Shown is the chemical profile of the compound with the highest-intensity peak among the 73 detected compounds, including retention time (RT), mass-to-charge ratio (*m*/*z*), mass, peak height, volume (Vol), and relative peak area percentage (Area %).

Comprehensive LC-MS profiling of the *A. maurorum* ethanol crude extract revealed a chemically diverse, intricate phytochemical profile (Figure 1A,B). A total of 73 chromatographic features were tentatively annotated based on accurate mass, retention behavior, and database/literature comparison. These annotations should be interpreted as putative assignments from crude extract profiling rather than as definitive structural confirmation. The detected metabolites were mainly classified as phenolic acids, glucosinolates, gallotannins, alkaloids, fatty acids, carotenoid derivatives, and *2-hexyldecanoic acid*-related compounds. Among the key sulfur-containing metabolites, 7-methylthioheptyl glucosinolate was prominent, a class known for its antimicrobial and chemopreventive properties [1,2,3]. Phenolic compounds, including 2,4,6-trihydroxybenzoic acid, β-glucogallin, and galloylglycerol, were also identified; these have been reported to exert antibacterial activity through mechanisms such as membrane destabilization and protein precipitation [4,5,6].

Furthermore, the detection of bergenin and elaeocarpidine indicates potential anticancer activity, as these compounds have been shown to induce apoptosis and inhibit proliferation in various cancer cell lines [8,9,10,11]. Notably, *2-hexyldecanoic acid* was identified as a major bioactive component (7.49% peak area), aligning with previous studies that describe *2-hexyldecanoic acid* derivatives as multifunctional compounds with antioxidant, antibacterial, and anticancer effects [8,9]. Overall, the LC-MS results identify 73 compounds (Supplementary Index Table S1), of which only 17 have a solid chemical foundation supporting the biological activities observed in this study (Table 1). We excluded the unknown compounds and compounds that did not show antibacterial and/or anticancer activities.

### 2.2. Antibacterial Activity

**Figure 2:**
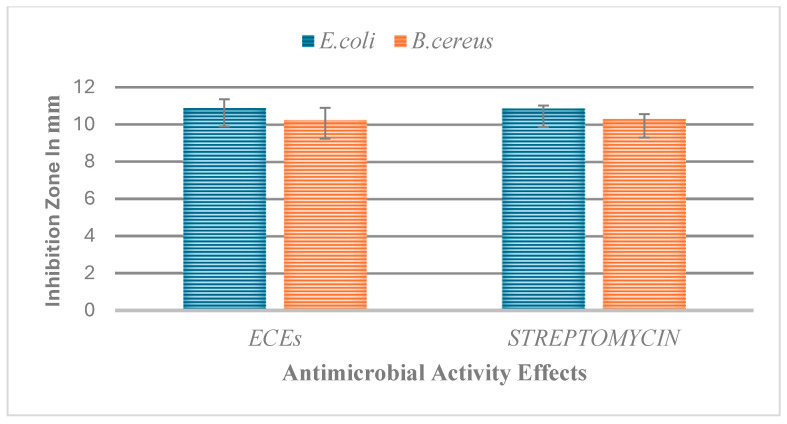
The antibacterial activity effects of ethanol crude extracted (ECEs) from *A. maurorum*.

The antibacterial activity of the *A. maurorum* ethanol crude extract was assessed against Gram-negative (*E. coli*) as well as Gram-positive (*B. cereus*) bacteria. Disk diffusion assays yielded clear, reproducible zones of inhibition for both strains. The extract exhibited inhibition zones measuring 10.9 ± 0.4 mm against *E. coli* and 10.2 ± 0.1 mm against *B. cereus*. These values can be compared to those of the positive control, streptomycin, under similar experimental conditions (Table 2). These results demonstrate that the extract possesses antibacterial activity against *E. coli and B. cereus* (Figure 2 and Table 2). The moderate inhibition observed (12–15 mm) suggests that the crude extract contains bioactive compounds at relatively low concentrations. Therefore, further purification and fractionation are recommended to isolate and concentrate the active constituents, thereby enhancing antimicrobial activity.

**Figure 3:**
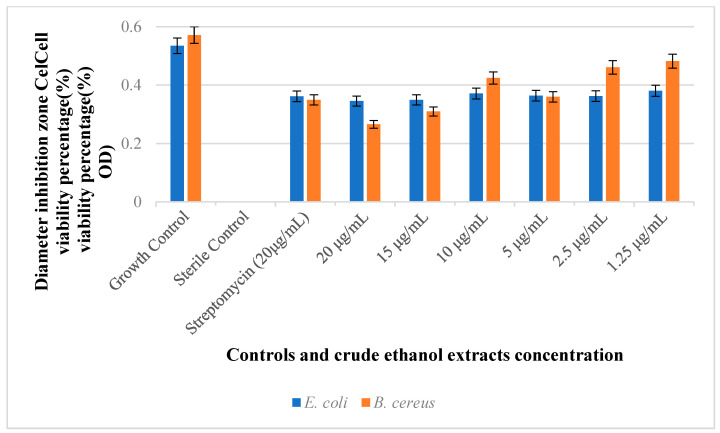
The MIC and disc diffusion assay results of ethanol extracted from *A. maurorum* against *E. coli* (Gram-negative) and *B. cereus* (Gram-positive). The extract demonstrated consistent, reproducible antibacterial effects comparable to those of the positive control, streptomycin. The results are expressed as ± standard deviation (SD) (*n* = 3).

The MIC results corroborated these findings by showing concentration-dependent inhibition of bacterial proliferation (Figure 3). The demonstrated antibacterial activity likely reflects the combined action of phenolic acids, fatty acids, gallotannins, and glucosinolate-derived metabolites, which are reported to exert synergistic effects through membrane disruption, enzyme inhibition, and interference with essential metabolic pathways [5,6,7,15,19]. The similar effectiveness observed against both Gram-negative and Gram-positive bacteria indicates that the extract contains bioactive constituents capable of penetrating and acting across bacterial cell wall architectures.

### 2.3. Cytotoxic and Selective Anticancer Activity

**Figure 4:**
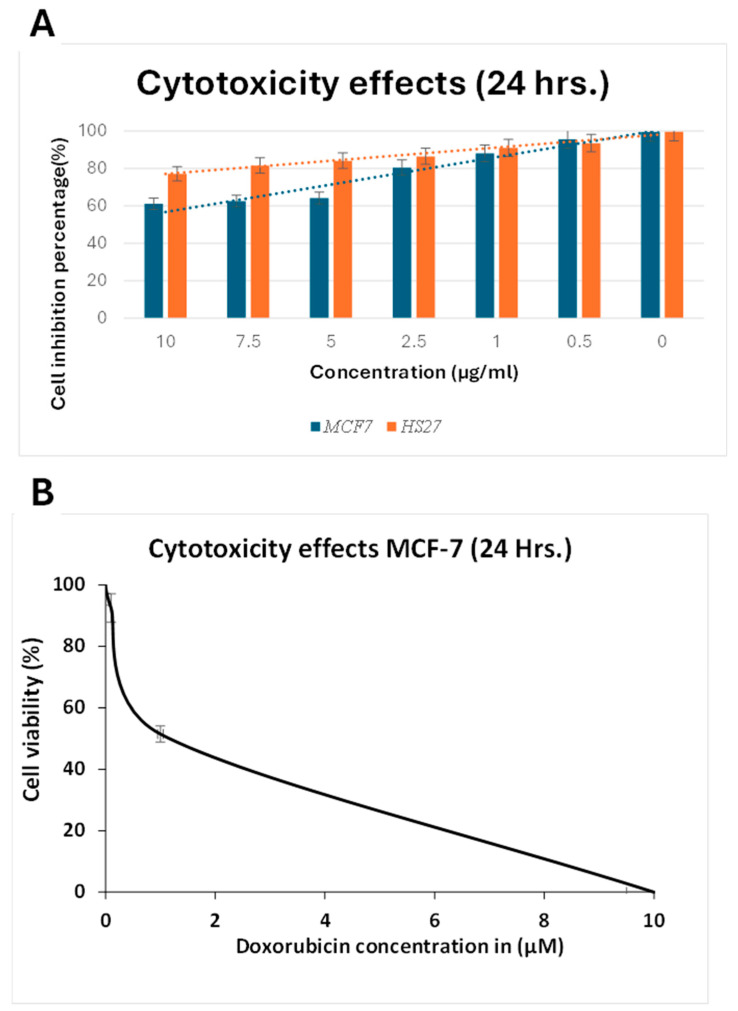
The cytotoxic effects (**A**) *A. maurorum* ethanol extract 24 h post-exposure, (**B**) Doxorubicin 24 h. The MTT assay results illustrate the viability of MCF-7 human breast cancer cells and Hs27 normal fibroblasts after 24 h of treatment with the ethanol extract and positive control (Doxorubicin). Cell viability is presented as a percentage relative, indicating early cytotoxic effects in cancer cells while showing minimal toxicity towards normal fibroblasts.

**Figure 5:**
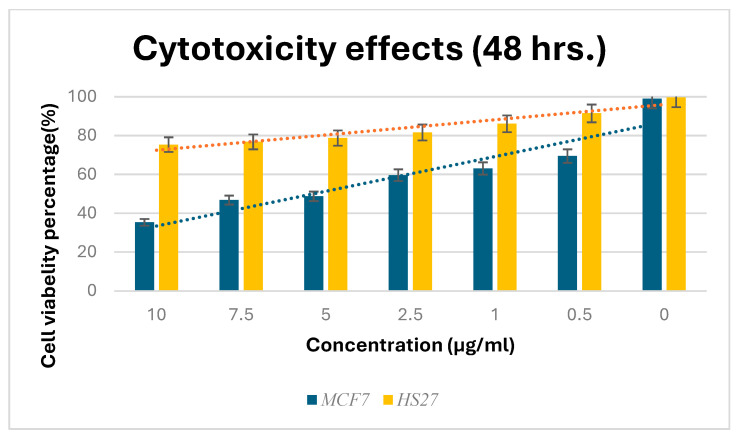
The time-dependent cytotoxicity of *A. maurorum* ethanol extract 48 h post-exposure. The MTT assay findings show a more pronounced decline in MCF-7 cell viability after 48 h of treatment, whereas Hs27 fibroblasts maintain comparatively higher viability. These results demonstrate increasing cytotoxicity over time and greater selectivity towards cancer. cells.

**Figure 6:**
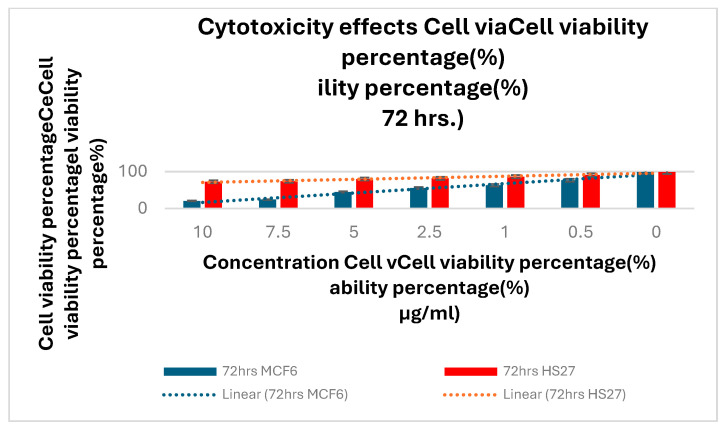
The selective anticancer activity of ethanol extracted from *A. maurorum* 72 h post-exposure. The MTT assay results after 72 h of treatment show a marked decrease in MCF-7 cell viability compared with Hs27 normal fibroblasts. The time-dependent reduction suggests cumulative activation of cancer cell death pathways, with data reported as ± SD (*n* = 3).

The pre-screening anticancer activity of the extract was evaluated using the MTT assay on MCF-7 human breast cancer cells and Hs27 normal fibroblasts at 24, 48, and 72 h. Treatment with the *A. maurorum* ethanol crude extract produced a clear time-dependent decrease in MCF-7 cell viability. The cytotoxic effect was minimal at 24 h, became more pronounced at 48 h, and reached its peak at 72 h, suggesting cumulative effects or the delayed activation of apoptotic pathways (Figure 4, Figure 5 and Figure 6).

Conversely, Hs27 fibroblasts maintained considerably higher viability at all time points, indicating selective cytotoxicity towards cancer cells. This selective profile is a key feature of effective anticancer agents. It aligns with previous studies on bergenin, 2-hexyldecanoic acid, and phenolic compounds, which preferentially affect cancer cells due *to* their heightened oxidative stress and altered metabolic states [8,9,10,11,20].

### 2.4. Apoptosis Assessment by Propidium Iodide (Pi) Staining

**Figure 7:**
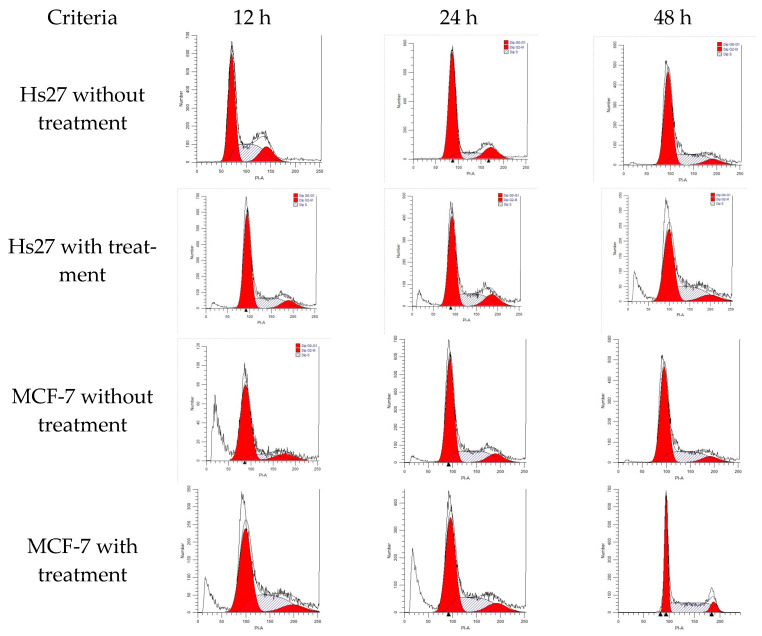
The induction of apoptosis in the MCF-7 cells by ethanol extracted from *A. maurorum*; propidium iodide (PI) staining of treated and untreated cells visualized by fluorescence microscopy. The treated MCF-7 cells display increased PI uptake, reflecting the loss of membrane integrity characteristic of late-stage apoptosis, while the Hs27 normal fibroblasts show minimal PI staining.

To further investigate the pre-screening mechanism of cell death, PI staining was used to evaluate membrane integrity. Fluorescence microscopy showed a substantial increase in PI-positive MCF-7 cells after treatment with the extract, indicating loss of membrane integrity consistent with late-stage apoptosis or secondary necrosis. By contrast, the untreated controls and treated Hs27 fibroblasts exhibited minimal PI uptake (Figure 7). The study findings showed distinct cell cycles between the normal human fibroblast cell line (Hs27) and human breast cancer cell line (MCF-7), following treatment time (12, 24, and 48 h). In the untreated Hs27, the G0/G1 phase showed the majority of cells (60–65%), the S phase at (18–20%), and G2/M at (9–11%). Unlike Hs27, the untreated MCF-7 cells were shown to be in G0/G1 (48–51%), S (28–30%), and G_2_/M (17–20%). This distribution reflects the high cycling activity characteristic of malignant cells.

Following the treated cell lines finding, Hs27 showed different cell cycle responses, with cells residing in the G0/G1 phase increasing to 78–80% after 48 h. However, the S phase and G_2_/M were decreased after 48 h. This pattern suggests that the treatment induces a G1 checkpoint, thereby suppressing DNA replication and cell cycle progression. Such G1 arrest is a common protective response in normal cells, often associated with activation of p53-dependent pathways and induction of senescence, rather than apoptosis.

In contrast, MCF-7 showed a different cell cycle response pattern compared to Hs27; G_2_/M phase rose from ~40% at 12 h to ~60% at 48 h. In the meantime, both G_0_/G_1_ and S phase fractions were decreased. Prolonged G2/M arrest in cancer cells often precedes apoptosis or mitotic catastrophe, underscoring the treatment’s cytotoxic potential.

These results indicate that apoptosis is a primary mechanism driving the observed cytotoxicity and align with previous reports showing that 2-hexyl-decanoic acid and bergenin promote apoptosis through mitochondrial dysfunction, reactive oxygen species generation, and the activation of intrinsic apoptotic pathways [8,9,10,11,21,22]. Our findings suggest that 2-hexyl-decanoic acid affects bioactivities among the 73 detected compounds, as it ranked among the five highest-intensity compounds (Figure 1B). However, these findings were consistent with a previous report showing that 2-hexyldecanoic acid has antibacterial and minimal anticancer activity (Table 1).

### 2.5. Molecular Docking Analysis

**Figure 8:**
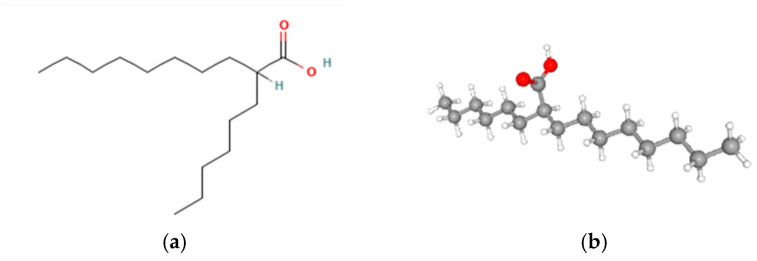
The predicted two-dimensional (**a**) and three-dimensional (**b**) binding interactions of 2-hexyl-decanoic acid (identified with www.pubchem.ncbi.nlm.nih.gov (accessed on 25 February 2025)).

A molecular docking analysis was performed to investigate the interaction between 2-hexyldecanoic acid and key bacterial target proteins, including DNA gyrase subunit A (GyrA), DNA gyrase subunit B (GyrB), and RNA polymerase subunit β (RpoB). The docking results demonstrated favorable ligand binding within the active sites of all three proteins (Figure 8 and Table 3). The predicted binding energies were −6.2 kcal/mol for GyrA, −6.7 kcal/mol for GyrB, and −7.0 kcal/mol for RpoB, indicating moderate binding affinity. Among the targets, the strongest interaction was observed with RpoB, suggesting a higher binding stability within the RNA polymerase active site.

The structural analysis of the docked complexes revealed that 2-hexyldecanoic acid adopts conformations that allow its long hydrophobic alkyl chain to interact extensively with nonpolar residues within the binding pockets. At the same time, the terminal carboxyl group is oriented toward polar residues, enabling potential hydrogen-bond interactions.

In the GyrA complex, the ligand was positioned within the quinolone-binding region, interacting with residues such as **Ser83, Asp87, Ala68, Ile90, and Val120**, primarily through hydrophobic and van der Waals interactions. In GyrB, the ligand occupied the ATP-binding pocket, forming interactions with **Asp73, Gly77, Ile78, Val120, and Arg136**, suggesting possible interference with ATP-dependent enzymatic activity. In RpoB, the ligand was localized within the rifampicin-binding region, interacting with **His526, Asp516, Ser531, Leu533, and Phe514**, potentially disrupting RNA polymerase function.

Overall, the interaction profiles were dominated by hydrophobic and van der Waals contacts, with limited hydrogen-bond contributions, consistent with the physicochemical properties of 2-hexyldecanoic acid. These findings suggest that the compound may exert antibacterial effects by inhibiting multiple enzymes involved in DNA replication and transcription. As molecular docking provides a predictive model, these results should be interpreted as supportive mechanistic evidence and require further experimental validation.

These interactions align with prior studies showing that phenolic and *2-hexyldecanoic acid*-derived compounds can inhibit key bacterial enzymes, thereby contributing to antibacterial effects at the molecular level [13,14,23,24].

### 2.6. In Silico Toxicity and Absorption, Distribution, Metabolism, and Excretion (ADME) Profiling

While the compound (cpd 60) showed the highest abundance among the 73 compounds detected by LC-MS, we selected 2-hexyldecanoic acid for in silico investigations based on previous research findings of potent antibacterial and anticancer activities (Figure 1B and Table 1). The in silico *toxicity* prediction indicated that *2-hexyldecanoic acid*, categorized as ProTox-3.0 moderate acute oral toxicity (median lethal dose = 250 mg/kg; GHS Class III), was predicted to be inactive for mutagenicity and carcinogenicity. Organ-specific estimates indicated probable immunotoxic and nephrotoxic effects at higher exposure levels (Table 4).

The in silico toxicity profiling of *2-hexyldecanoic acid* was carried out using ProTox-3.0. The compound was estimated to contain an oral median lethal dose of 250 mg/kg, about toxicity class III under the Globally Harmonized System (GHS), signifying moderate acute oral toxicity. The projection was backed by 100% average similarity and model accuracy, indicating high confidence in the model’s applicability domain. Organ-oriented toxicity models projected inactive hepatotoxicity, cardiotoxicity, and neurotoxicity, with probabilities exceeding 0.67. Notably, nephrotoxicity (probability = 0.55) and respiratory toxicity (probability = 0.56) were estimated to be active, albeit with moderate confidence, suggesting likely organ-specific risks under specific exposure conditions.

The assessment of toxicological endpoints indicated that 2-hexyldecanoic *acid* is predicted to be inactive for carcinogenicity and mutagenicity, supporting a relatively favorable long-term genotoxic safety profile. In contrast, the compound showed predicted immunotoxicity (probability = 0.95) and clinical toxicity (probability = 0.56), suggesting potential immune modulation and systemic effects that warrant further experimental investigation. Additionally, blood–brain barrier permeability was predicted as active (probability = 0.59), signifying possible exposure to the central nervous system.

An analysis of the Tox21 nuclear receptor and stress–response pathways indicated no significant activation across all evaluated targets, including androgen, estrogen, aryl hydrocarbon, and PPAR-γ receptors, as well as oxidative stress and p53 signaling pathways. These results indicate a low likelihood of endocrine disruption or stress-mediated toxicity.

For molecular initiating events (MIEs), *2-hexyldecanoic acid* was predicted to be active against thyroid hormone receptor alpha (THRα) and NADH-quinone oxidoreductase (NADHOX), suggesting potential effects on thyroid hormone signaling and redox-related biological processes. All assessed cytochrome P450 isoforms were predicted to be inactive, indicating a low risk of inhibiting major metabolic enzymes. Despite these promising computational predictions, it is important to emphasize that in silico toxicity assessment provides only theoretical estimations based on predictive models and does not substitute for experimental validation. Therefore, the safety profile of 2-hexyldecanoic acid should be confirmed through in vivo toxicological studies, including acute and repeated-dose assessments, to establish its dose-dependent effects and long-term safety.

Overall, these in silico results suggest that *2-hexyldecanoic acid* has moderate acute toxicity and a generally favorable genotoxic and carcinogenic profile, while indicating potential immunotoxic, renal, and respiratory effects. These findings underscore the need for dose-dependent, long-term experimental research to validate safety (Table 4).

The pharmacokinetic evaluation of *2-hexyldecanoic acid* using SwissADME indicated favorable drug-like properties, including high gastrointestinal absorption, blood–brain barrier permeability, and full compliance with Lipinski, Ghose, Veber, Egan, and Muegge rules, with no predicted cytochrome P450 inhibition (Table 5). These results support its potential as an orally bioavailable compound, while emphasizing the need for dose optimization and in vivo safety studies [9,12,18]. The in silico *ADME analysis revealed that 2-hexyldecanoic acid* has a molecular weight of 294.39 g/mol, within the optimal range for oral bioavailability. It contains four hydrogen bond acceptors, two hydrogen bond donors, and a topological polar surface area of 66.76 Å^2^, reflecting a favorable balance between polarity and membrane permeability. High gastrointestinal absorption supports oral administration potential, and blood–brain barrier permeability aligns with its moderate lipophilicity (consensus logP ≈ 3.2). The fraction of sp^3^ carbons (0.59) and 10 rotatable bonds signify moderate molecular flexibility, which might impact binding interactions and overall bioavailability.

The drug-likeness evaluation indicated that *2-hexyldecanoic acid* fully satisfies the Lipinski, Ghose, Veber, Egan, and Muegge criteria with no violations, supporting its classification as a drug-like molecule. The predicted bioavailability score of 0.55 suggests a reasonable probability of systemic exposure following oral administration. Notably, the compound triggered no pan-assay interference (PAINS) or Brenk structural alerts, indicating a low risk of assay artifacts or inherent structural toxicity.

Metabolic predictions showed that *2-hexyldecanoic acid* does not inhibit major cytochrome P450 isoforms (CYP2C19, CYP1A2, CYP2C9, CYP2D6, and CYP3A4), implying a low potential for drug–drug interactions. It was also predicted to be a non-substrate of P-gp, minimizing the risk of efflux-mediated reductions in bioavailability.

Collectively, the in silico *ADME and drug-likeness profiles suggest that 2-hexyldecanoic acid* exhibits favorable oral absorption, adequate permeability, low metabolic liability, and strong drug-like properties, thereby bolstering its further evaluation as a bioactive natural compound (Table 5).

## 3. Discussion

The current results indicate that the pre-screening of the ethanol crude extract of *A. maurorum* exhibits both antibacterial and anticancer properties, corroborated by supporting experimental and computational data. The antibacterial activity observed against Gram-positive and Gram-negative strains is likely due to the synergistic effects of several phytochemical groups identified via LC-MS, including glucosinolates, phenolic acids, gallotannins, and fatty acids, which are recognized for their ability to disrupt bacterial membranes and inhibit critical enzymes [3,6,7,25,26]. Phenolic acids and gallotannins can destabilize bacterial membranes and precipitate intracellular proteins, whereas glucosinolates and fatty acids impair membrane stability and interfere with vital enzymatic functions [3,6,7,21,24,27,28,29]. Furthermore, molecular docking studies indicate that *2-hexyldecanoic acid* may enhance antibacterial activity by targeting essential bacterial enzymes involved in DNA replication and transcription, like RNA polymerase and DNA gyrase [13,14,22,23,24,30].

The pronounced selective cytotoxicity seen in the MTT assays against MCF-7 breast cancer cells is particularly significant. Cancer cells exhibit heightened oxidative stress, disrupted mitochondrial function, and aberrant survival signaling, making them more vulnerable to apoptosis triggered by phytochemicals [20,22,24,25]. *2-hexyldecanoic acid* and bergenin derivatives have been shown to promote apoptosis by inducing mitochondrial membrane depolarization, increasing reactive oxygen species production, and regulating the NF-κB and MAPK pathways in malignant cells [8,10,11,21,30,31,32]. The elevated PI uptake detected in the treated MCF-7 cells aligns with these processes, reinforcing apoptosis as the primary mechanism of cell death.

Combining in silico toxicity and ADME analyses adds an extra dimension of translational significance. While *2-hexyldecanoic acid* was predicted to exhibit moderate acute toxicity and potential immunotoxic or nephrotoxic effects, these findings should be evaluated in the context of dose-dependent exposure and are frequently reported for bioactive plant-derived compounds [9,23,28,33,34,35]. The predicted safety profile, together with the observed antibacterial and selective anticancer activity, suggests that 2-hexyldecanoic acid represents a promising lead compound for further pharmacological investigation. Notably, the favorable pharmacokinetic characteristics of 2-hexyldecanoic acid—including high gastrointestinal absorption, the absence of cytochrome P450 inhibition, and adherence to multiple drug-likeness parameters—support its potential for further development and optimization [9,12,18,21,34,36,37,38]. Although crude extracts provide a holistic representation of plant bioactivity, further fractionation and identification of individual bioactive compounds are necessary to precisely elucidate the mechanisms of action and minimize variability [39]. Nevertheless, the LC–MS data in this study should be interpreted as a preliminary metabolite profiling of a crude extract. Since full analytical validation, authentic standard confirmation, and targeted MS/MS verification were not performed for all annotated constituents, these assignments remain tentative and should be confirmed in future bioactivity-guided fractionation studies.

In general, integrating phytochemical characterization, in vitro bioassays, molecular docking, and in silico safety profiling reinforces the mechanistic understanding of the observed antibacterial and anticancer activities. It reduces the likelihood that these effects are nonspecific or artefactual [16,18,38,40,41].

## 4. Materials and Methods

### 4.1. The Plant Material, Extraction, and Liquid Chromatography–Mass Spectrometry (LC-MS) Analysis

*Alhagi mauroru* was purchased from the BAX herbalist shop as the plant was collected from Dostaka village, Duhok mountain, Duhok province, Iraq, and identified by the National Herbarium of Iraq (BAG) with the voucher number (BAG/Ref. 1629). Then, the plant was subjected to Soxhlet extraction with ethanol to maximize the recovery of both polar and semi-polar phytochemicals. A total of 20 g of air-dried, powdered plant material was subjected to solvent extraction. The final crude extract yielded 1.82 g (representing a percentage yield of 9.1% based on the dry weight of the starting material). The resulting crude extract was filtered, concentrated under reduced pressure, and stored at 4 °C until further use [42]. The Agilent 1260 UPLC/6540 Q-TOF with an SB-C18 column (150 mm × 4.6 mm × 5 µL) was used to analyze the chemicals in the extracts. Methanol (solvent A) and water (solvent B) made up the solvent gradient. The column was kept at 30 °C, and the samples were eluted in electrospray ionization (ESI) mode at a mobile phase flow rate of 1 mL/min. The gradient elution method for ionization mode: 0–25 min, 20–100% solvent A; 25–30 min, 100–0% solvent A. Chromatographic separation was achieved using a reversed-phase C18 column under gradient elution conditions optimized to improve peak resolution of compounds with varying polarity. Repeat injections assessed system suitability, and only reproducible, well-defined peaks were included in the analysis. Although the applied LC conditions provided reasonable separation of the major constituents, complete baseline resolution of all compounds in the crude extract cannot be guaranteed due to the matrix’s complexity. Compound identification was achieved by comparing accurate mass (*m*/*z*) and retention times (RTs) with tentative assignments based on the published literature and established reference databases (PubChem and METLIN). For biological interpretation, only compounds previously documented in peer-reviewed studies were included to minimize the risk of over-annotation [43]. LC–MS data acquisition was performed in untargeted profiling mode, and metabolite annotation was based on accurate mass, retention time, isotope distribution pattern, and database matching against METLIN and PubChem, supported by literature reports. A mass tolerance threshold of ±5 ppm was applied during tentative annotation where applicable. To improve confidence and reduce false-positive assignments, blank runs and replicate injections were included, and only reproducible peaks consistently detected across replicate analyses were retained for interpretation. Peak areas were used to estimate relative abundance via normalization and do not represent absolute quantification. Because authentic reference standards were not analyzed for all detected metabolites, the reported compound assignments should be considered putative rather than definitive identifications, corresponding to MSI level 2–3 annotations. However, MS/MS confirmation with authentic standards was not performed in the present untargeted screening study.

The relative area percentage (Area %) of each constituent was calculated following the peak area normalization method described by [44] as shown in the equation below:

$$Area\;\%=\left(\frac{Peak\;area\;of\;target\;compound\;}{Total\;peak\;of\;all\;detected\;compounds}\right)\;x\;1$$

### 4.2. Antibacterial Activity Assays

The antibacterial activity of the ethanol crude extract was assessed using the disk diffusion assay against Escherichia coli (Gram-negative) and Bacillus cereus (Gram-positive). Sterile paper disks were soaked with definite concentrations of the extract and placed on agar plates pre-inoculated with bacterial strains. Streptomycin served as a positive control, and disks treated with solvent alone acted as negative controls. Following incubation at 37 °C for 18–24 h, the zones of inhibition were measured in millimeters (mm). All the experiments were carried out in triplicate, and the results were reported as mean ± standard deviation (SD) [45].

The minimum inhibitory concentration (MIC) values were determined by broth microdilution in 96-well plates. Serial dilutions of the extract were prepared and inoculated with standardized bacterial suspensions under the same incubation conditions. The MIC was defined as the lowest concentration at which no visible bacterial growth was noted [45,46].

### 4.3. Cell Culture and Cytotoxic 3-(4,5-Di Methyl Thiazol-2-Yl)-2,5-Diphenyltetrazolium Bromide (Mtt) Assay

MCF-7 human breast adenocarcinoma cells and Hs27 human dermal fibroblasts were maintained in complete growth medium under standard conditions (37 °C, 5% carbon dioxide). The cells were plated in 96-well plates and allowed to attach overnight before treatment. The extract was added at predetermined concentrations, and the cells were incubated for 24, 48, and 72 h. Cell viability was assessed using the 3-(4,5-dimethylthiazol-2-yl)-2,5-diphenyltetrazolium bromide (MTT) assay, which evaluates the mitochondrial conversion of a tetrazolium salt into formazan crystals. Absorbance was read spectrophotometrically, and viability was calculated as a percentage compared to the untreated control cells [46]. Doxorubicin was used as a positive control, and the cells were incubated for 24 h. The results were assessed and analyzed as described by [47].

### 4.4. Apoptosis Assessment via Propidium Iodide (Pi) Staining

To evaluate late-stage apoptosis and membrane integrity, both treated and untreated cells were stained with propidium iodide (PI). The cells were then analyzed by flow cytometry (FACSAria II from Becton, Dickinson, and Company, San Jose, CA, USA), which was used to identify PI-positive nuclei as non-viable or undergoing apoptotic or necrotic death [42].

### 4.5. Molecular Docking Studies

A molecular docking analysis was performed to investigate the potential interactions between the selected phytochemicals identified from LC–MS profiling and key bacterial target proteins, including DNA gyrase subunit A (GyrA), DNA gyrase subunit B (GyrB), and RNA polymerase subunit β (RpoB). The three-dimensional crystal structures of the target proteins were retrieved from the Protein Data Bank (PDB) and prepared by removing water molecules, co-crystallized ligands, and non-essential ions. Polar hydrogen atoms were added, and Kollman charges were assigned using AutoDock Tools (ADT) Vina 1.2.x.

The ligand, 2-hexyldecanoic acid, was obtained from the PubChem database and energy-minimized before docking. Gasteiger charges were assigned, and rotatable bonds were defined to allow conformational flexibility.

Docking simulations were carried out using AutoDock (Vina 1.2.x) with the Lamarckian Genetic Algorithm (LGA). A grid box was defined to encompass the active-site region of each protein, with appropriate dimensions and grid spacing to ensure complete coverage of the binding pocket. Default docking parameters were applied, including a population size of 150, a maximum of 2.5 × 10^6^ energy evaluations, and 10 independent docking runs per ligand.

The best docking pose for each protein–ligand complex was selected based on the lowest binding energy and cluster consistency. Binding interactions, including hydrogen bonds, hydrophobic contacts, and van der Waals interactions, were analyzed and visualized using Discovery Studio Visualizer 2022 (BIOVIA, San Diego, CA, USA) and PyMOL 2.5 (Schrödinger, LLC., New York, NY, USA). As molecular docking provides a predictive model of ligand–protein interactions, the results were interpreted as preliminary mechanistic insights and require further experimental validation [42].

### 4.6. In Silico Toxicity and Absorption, Distribution, Metabolism, and Excretion (ADME) Prediction

An in silico toxicity assessment was performed by utilizing ProTox-3.0 to predict acute oral toxicity, organ-specific toxic effects, carcinogenic potential, and mutagenicity. Pharmacokinetic and drug-likeness properties were analyzed using Swiss ADME, including gastrointestinal absorption, cytochrome P450 inhibition, blood–brain barrier permeability, and adherence to established drug-likeness criteria [42].

## 5. Conclusions

This work highlights that the crude ethanol extract of *A. maurorum* exhibits both antibacterial and anticancer effects, as corroborated by chemical profiling, biological assays, and computational analyses. The recognition of *2-hexyldecanoic acid* as a principal active component, along with its promising in silico pharmacokinetic profile, underscores the plant’s potential as a source of lead compounds for further exploration. Subsequent research should emphasize in vivo validation, the purification of bioactive constituents, and comprehensive mechanistic studies to enhance its translational applicability.

## Figures and Tables

**Table 1:** Compounds found in crude ethanol extracts with LCMS (17 compounds were shown to have bioactivity effects from previous research).

No.	Compound	Formula	Antibacterial Activity	Anticancer Activity	Reference(s)
**1**	7-Methylthioheptyl glucosinolate	C15 H29 N O9 S3	Possible	Possible	[1,2]
**2**	Tartaric acid	C4 H6 O6	Possible	No	[3]
**3**	2,4,6-Trihydroxybenzoic acid	C7 H6 O5	Yes	Possible	[3]
**4**	β-Glucogallin	C13 H16 O10	Yes	Possible	[4]
**5**	1-O-Galloylglycerol	C10 H12 O7	Yes	Possible	[5]
**6**	Bergenin	C14 H16 O9	Yes	Yes	[6,7]
**7**	4-Ketolutein D (carotenoid derivative)	C40 H54 O3	Possible	Possible	[8]
**8**	2-hexyldecanoic acid	C16 H32 O2	Strong (Gram-positive bacteria, fungi)	Strong (apoptosis, anti-angiogenesis, cell cycle arrest)	[9,10]
**9**	Undecylbenzenesulfonic acid	C17 H28 O3 S	Yes	No	[11,12,13]
**10**	2-Dodecylbenzenesulfonic acid	C18 H30 O3 S	Yes	No	[14]
**11**	Elaeocarpidine	C17 H21 N3	Yes	Yes	[15]
**12**	PI-Cer t18:0/20:0(2OH)	C44 H88 N O13 P	Indirect	Yes	[16]
**13**	6′-Hydroxysiphonaxanthin	C40 H56 O5	Possible	Yes	[17]
**14**	7Z,10Z-Octadecadienoic acid (PUFA)	C18 H32 O2	Mild (membrane disruption)	Possible (weak/modulatory)	[18]
**15**	2-hexyl-decanoic acid (branched FA)	C16 H32 O2	Likely antibacterial	Minimal	[19]
**16**	5Z-Octadecenoic acid (MUFA)	C18 H34 O2	Mild	Minimal/weak	[18]
**17**	14-Methylheptadecanoic acid (branched SFA)	C18 H36 O2	Possible	No clear evidence	[18]

**Table 2:** The sizes of the inhibition zones from the disk diffusion assay.

	*A. maurorum*	Streptomycin
*E. coli*	10.9 ± 0.4	10.8 ± 0.6
*B. cereus*	10.2 ± 0.1	10.3 ± 0.2

**Table 3:** The molecular docking at the active sites of GyrA, GyrB, and RpoB. The docking results reveal stable hydrogen bonds and hydrophobic interactions, supporting a potential enzyme-inhibition mechanism underlying the observed antibacterial activity.

Protein	Ligand	Binding Affinity (kcal/mol)	Binding Region	Key Interacting Residues	Interaction Type
GyrA 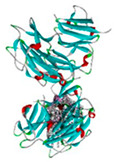	2-hexyldecanoic acid	−6.2	Quinolone-binding region	Ser83, Asp87, Ala68, Ile90, Val120	Hydrophobic, van der Waals, possible H-bond
GyrB 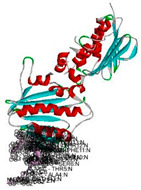	2-hexyldecanoic acid	−6.7	ATP-binding pocket	Asp73, Gly77, Ile78, Val120, Arg136	Hydrophobic, van der Waals, possible H-bond
RpoB 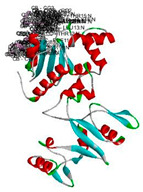	2-hexyldecanoic acid	−7.0	Rifampicin-binding region	His526, Asp516, Ser531, Leu533, Phe514	Hydrophobic, van der Waals, possible H-bond

Residues are predicted based on docking pose visualization within known active-site regions. Interactions are primarily hydrophobic, driven by the ligand’s aliphatic structure, with limited hydrogen-bonding from the carboxylic acid group.

**Table 4:** A summary of the predicted toxicities.

Category	Endpoint	Prediction	Probability
Acute Toxicity	Oral median lethal dose (mg/kg)	250	—
	Toxicity Class (GHS)	Class III	—
Organ Toxicity	Hepatotoxicity	Inactive	0.83
	Neurotoxicity	Inactive	0.83
	Nephrotoxicity	Active	0.55
	Respiratory toxicity	Active	0.56
	Cardiotoxicity	Inactive	0.67
Toxicological Endpoints	Carcinogenicity	Inactive	0.76
	Mutagenicity	Inactive	0.65
	Immuno-toxicity	Active	0.95
	Cytotoxicity	Inactive	0.88
	Blood–brain barrier permeability	Active	0.59
	Eco-toxicity	Inactive	0.63
	Clinical toxicity	Active	0.56
Tox21 Pathways	Nuclear receptor signaling	Inactive	0.71–0.99
	Stress response pathways	Inactive	0.59–0.98
Molecular Initiating Events	THRα	Active	0.56
	NADHOX	Active	0.70
Metabolism	CYP450 inhibition (CYP1A2, 2C9, 2D6, 3A4, 2E1)	Inactive	0.53–1.00

**Table 5:** A summary of the ADME prediction.

Category	Parameter	Predicted Value
Physicochemical properties	Molecular weight (g/mol)	294.39
	Molecular formula	C_17_H_26_O_4_
	Hydrogen bond acceptors	4
	Hydrogen bond donors	2
	Topological polar surface area (Å^2^)	66.76
	Rotatable bonds	10
	Fraction Csp^3^	0.59
Lipophilicity	Consensus logP	~3.2
Absorption & distribution	Gastrointestinal absorption	High
	Blood–brain barrier permeability	Yes
	P-gp substrate	No
Metabolism	CYP1A2 inhibition	No
	CYP2C19 inhibition	No
	CYP2C9 inhibition	No
	CYP2D6 inhibition	No
	CYP3A4 inhibition	No
Drug-likeness	Lipinski violations	0
	Ghose violations	0
	Veber violations	0
	Egan violations	0
	Muegge violations	0
	Bioavailability score	0.55
Medicinal chemistry alerts	Pan-assay interference compounds alerts	0
	Brenk alerts	0
	Synthetic accessibility	Moderate

## Data Availability

The original contributions presented in this study are included in the article/Supplementary Material. Further inquiries can be directed to the corresponding authors.

## Supplementary Materials

The following supporting information can be downloaded at https://www.mdpi.com/article/10.3390/ijms27114766/s1.
